# Validation of the modified radiographic union score for tibia fractures (mRUST) in murine femoral fractures

**DOI:** 10.3389/fendo.2022.911058

**Published:** 2022-08-03

**Authors:** Vincent J. Alentado, Adam M. Knox, Caio A. Staut, Anthony C. McGuire, Joseph R. Chitwood, Sarah L. Mostardo, Mustufa Z. Shaikh, Rachel J. Blosser, Usashi C. Dadwal, Tien-Min Gabriel Chu, Christopher D. Collier, Jiliang Li, Ziyue Liu, Melissa A. Kacena, Roman M. Natoli

**Affiliations:** ^1^ Department of Neurological Surgery, School of Medicine, Indiana University, Indianapolis, IN, United States; ^2^ Department of Orthopaedic Surgery, School of Medicine, Indiana University, Indianapolis, IN, United States; ^3^ Department of Biomedical Sciences and Comprehensive Care, School of Dentistry, Indiana University, Indianapolis, IN, United States; ^4^ Department of Biology, Indiana University, Purdue University, Indianapolis, IN, United States; ^5^ Department of Biostatistics and Health Data Science, School of Public Health, Indiana University, Indianapolis, IN, United States; ^6^ Richard L. Roudebush VA Medical Center, Department of Veterans Affairs, Indianapolis, IN, United States

**Keywords:** fracture healing, bone healing, fracture biomechanics, histomorphometry, micro computed tomography, radiographic union score for tibial fractures

## Abstract

Bony union is a primary predictor of outcome after surgical fixation of long bone fractures. Murine models offer many advantages in assessing bony healing due to their low costs and small size. However, current fracture recovery investigations in mice frequently rely on animal sacrifice and costly analyses. The modified Radiographic Union Score for Tibia fractures (mRUST) scoring system is a validated metric for evaluating bony healing in humans utilizing plain radiographs, which are relatively inexpensive and do not require animal sacrifice. However, its use has not been well established in murine models. The aim of this study was to characterize the longitudinal course of mRUST and compare mRUST to other conventional murine fracture analyses. 158 mice underwent surgically created midshaft femur fractures. Mice were evaluated after fracture creation and at 7, 10, 14, 17, 21, 24, 28, 35, and 42 days post-injury. mRUST scoring of plain radiographs was performed by three orthopaedic surgeons in a randomized, blinded fashion. Interrater correlations were calculated. Micro-computed tomography (μCT) was analyzed for tissue mineral density (TMD), total callus volume (TV), bone volume (BV), trabecular thickness, trabecular number, and trabecular separation. Histomorphometry measures of total callus area, cartilage area, fibrous tissue area, and bone area were performed in a blinded fashion. Ultimate torque, stiffness, toughness, and twist to failure were calculated from torque-twist curves. A sigmoidal log-logistic curve fit was generated for mRUST scores over time which shows mRUST scores of 4 to 6 at 7 days post-injury that improve to plateaus of 14 to 16 by 24 days post-injury. mRUST interrater correlations at each timepoint ranged from 0.51 to 0.86, indicating substantial agreement. mRUST scores correlated well with biomechanical, histomorphometry, and μCT parameters, such as ultimate torque (r=0.46, p<0.0001), manual stiffness (r=0.51, p<0.0001), bone percentage based on histomorphometry (r=0.86, p<0.0001), cartilage percentage (r=-0.87, p<0.0001), tissue mineral density (r=0.83, p<0.0001), BV/TV based on μCT (r=0.65, p<0.0001), and trabecular thickness (r=0.78, p<0.0001), among others. These data demonstrate that mRUST is reliable, trends temporally, and correlates to standard measures of murine fracture healing. Compared to other measures, mRUST is more cost-effective and non-terminal. The mRUST log-logistic curve could be used to characterize differences in fracture healing trajectory between experimental groups, enabling high-throughput analysis.

## Introduction

Approximately 1.1 million human bone fractures are treated annually in the United States (US). In fact, long bone fractures comprise 10% of all non-fatal injuries and incur an estimated inpatient expenditure of $10.4 billion annually in the US (1). Fracture union is a key component in patients’ post-injury health-related quality of life. Unfortunately, delayed union and non-union are encountered in approximately 5% of all fractures, or 55,000 fractures annually in the US alone (2). These adverse outcomes are associated with increased healthcare costs, worsened quality of life, and indirect costs associated with loss of productivity due to long recovery periods for patients. Given the enormous physical and socioeconomic impact of delayed union and nonunion after long bone fractures, it is critical to obtain an improved understanding of the molecular and physiologic basis of fracture healing. This knowledge will help drive novel therapeutic strategies that can enhance fracture union and patient outcomes.

Whether assessing fracture healing in mouse models or human patients, radiographic imaging is pivotal. Computed tomography (CT) scanning provides quantitative, three-dimensional measurements of both the structure and mineralization of the fracture callus. In fact, numerous studies have demonstrated that CT-based measurements directly correlate with callus stiffness and strength (3, 4). In the realm of small animal studies, μCT is often used to provide superior resolution compared to other CT modalities. *In vivo* μCT allows for the generation of longitudinal data, and studies have identified protocols for non-invasive monitoring of rodent fracture healing (5–8). However, compared to CT imaging, plain radiographs offer lower costs, shorter anesthetic times, less radiation, and simplification of technique. Despite these advantages, McClelland et al. (9) found that mechanical stiffness estimates for tibial fractures were inaccurate when based solely on the general appearance of plain radiographs in human patients. Given the lack of a consistent, effective method of classifying bony union and stage of healing in tibial fractures, Wheland et al. (10) developed the Radiographic Union Score for Tibia fractures (RUST scores) for clinical use, which was subsequently modified. The modified RUST (mRUST) assigns values of 1 to4 for each cortex. Compared to RUST, the mRUST score has higher intra-class correlation coefficients (ICC) with scores ranging from 0.86 to 0.96 (11, 12). Additionally, mRUST scores have been shown to correlate with mechanical stiffness estimates (4).

Despite the success of mRUST in human studies, there is no accepted methodology for utilizing plain radiographs to assess fracture healing in mouse models. The standard measures of fracture healing in mice include biomechanical testing, μCT analysis, and histomorphometric analysis, which are associated with significant time and costs (13). Should mRUST scoring show consistent reflection of the more traditionally accepted measures, it could support a new avenue for fracture assessment in mice that is cheaper, faster, and more clinically translatable than current standard measures, making it an appealing prospect for high throughput analysis. We hypothesized that mRUST scoring would quantitatively capture fracture healing in a mouse model of surgically created femur fractures and that interrater reliability would be adequate. We further hypothesized that mRUST would correlate well with biomechanical properties, μCT measures, and histomorphometric analyses.

## Methods

All animal procedures were performed with prior approval from Indiana University School of Medicine Institutional Animal Care and Use Committee. All experiments were performed in compliance with NIH guidelines on the use and care of laboratory and experimental animals. Three month old male, C57BL6/J mice were purchased from Jackson Laboratories (Bar Harbor, ME) and housed in the Indiana University School of Medicine Laboratory Animal Resource Center under a 12-h light, 12-h dark cycle for 6 weeks. Food and water were provided ad libitum.

### Fracture induction

Mice were anesthetized using 1.0% isoflurane for induction and 0.6-0.8% for maintenance, at 1000 mL/minute utilizing a SomnoSuite Low-Flow Anesthesia System (Kent Scientific). Analgesia was provided by 1.0mg/kg of buprenorphine sustained-release subcuticular fashion pre-operatively. The right leg was shaved and scrubbed with betadine followed by ethanol (triplicate scrubs). All instruments and implants were autoclaved before use. A surgically created mid-shaft femur fracture was utilized as previously described (3). In brief, utilizing sterile technique, a 1-cm incision was made over the anterolateral distal femur. The patella was laterally dislocated to expose the distal femur. With the knee flexed, a 25-gauge hypodermic needle was passed into the intramedullary canal, entering between the condyles and being inserted retrograde. The needle was then pre-bent over the greater trochanter for post-mortem removal. Finally, the needle was then cut as flush to the cartilage as possible in the femoral notch, which is not in the weight bearing area of the joint (ie, not between the femoral condyles and tibial plateaus). We have not noted any issues with mouse mobility/use of the injured limb after the procedure or observed osteoarthritis of the knee in the time frame studied. As all fractures healed, it is unlikely that the experimental results are affected by retrograde placement of the needle. Finally, a mid-shaft transverse fracture was created within same incision using a dental wire cutter (Hu-Friedy Manufacturing, Chicago, IL). An acceptable fracture pattern, defined as a simple, transverse, mid-diaphyseal fracture without comminution or shortening, was verified under direct visualization and immediate post-operative X-ray. The extensor mechanism was repositioned, and the fascia closed with 3–0 Vicryl suture (Ethicon, Inc., Somerville, NJ). The mice were allowed free, unrestricted weight bearing for up to 42 days post-injury. Animals were randomly assigned to the different time points and were housed under standard conditions (2-5 animals/cage). Mice were evaluated at the following intervals: 7, 10, 14, 17, 21, 24, 28, 35, and 42 days post-injury.

### X-ray imaging

**Figure 1:**
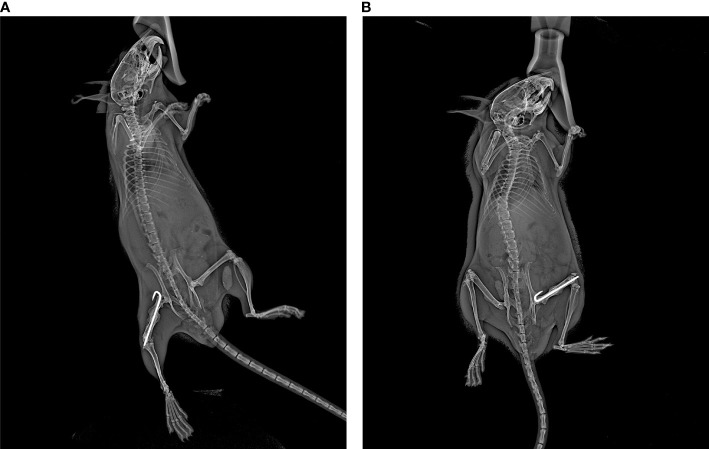
Example of mouse positioning for anteroposterior **(A)** and lateral **(B)** radiographs.

Anteroposterior (AP) and lateral X-ray imaging at the aforementioned time points were obtained using a Kubtec Xpert80 X-ray system (Kubtec Medical Imaging, Stratford, CT). All images were taken using a set of standardized imaging parameters to ensure direct comparisons within and across specimens. AP radiographs were obtained by placing the mouse on its left (non-surgical) hip. A small piece of tape was used to gently pull the surgical leg laterally and downwards while ensuring that the tail was not overlapping the femur (**Figure 1A**). Lateral radiographs were obtained by placing the mouse prone and splaying both hindlimbs, while again ensuring the tail was not overlapping the femur (**Figure 1B**). Mice were anesthetized using inhaled isoflurane (SomnoSuite settings were 1.0% for induction and 0.1-0.3% for maintenance, at 1000mL/minute; a separate SomnoSuite unit was used during the brief X-ray imaging, which was set at 3.0% at 100mL/minute), and X-ray was acquired over an eight second interval at 45mV.

### mRUST scoring

Three orthopaedic surgeons determined an mRUST score for each X-ray image. The images were presented in random order, and surgeons were blinded to the time of radiograph. The mRUST assigns an integer score to each cortex imaged on the AP (medial and lateral cortices) and lateral (anterior and posterior cortices) X-rays as follows: 1 = no healing; 2 = callus present, no bridging; 3 = bridging callus, fracture line visible; 4 = bridging callus with no fracture line visible. The scores for all 4 cortices were summed to provide a final score ranging from 4 (not healed) to 16 (maximally healed). Mean mRUST scores were calculated for each specimen at each time point, and interrater correlations were calculated.

### Euthanasia and sample storage

Mice were euthanized at the indicated time points using carbon dioxide gas and cervical dislocation. Both the fractured and unfractured femora were harvested and cleaned of subcutaneous tissue and muscle before analysis. The intramedullary needle was removed using a needle driver to facilitate histologic sectioning and limit metal artifact during μCT analysis. Femora designated for histomorphometry and μCT were fixed in 10% neutral buffered formalin for 48 hours and then rinsed and stored in 70% ethanol at 4°C. Femora designated for μCT and biomechanical analysis were wrapped with gauze soaked in deionized water and stored at -20°C.

### Micro-computed tomography imaging

Analyses were conducted after all mice were euthanized and all specimens were treated at the same time to minimize batch to batch variation. For μCT analyses, the scanner was calibrated using two phantom rods 2mm in diameter, at concentrations of 0.25 and 0.75 g/cm^3^. The long axis of each femur was aligned with the vertical axis of the scanner. Femurs were imaged using a Bruker SkyScan 1172 desktop imaging system with X-ray tube voltage 59kV, intensity 167μA, rotation step 0.7°, integration time 885ms, and voxel size 9.83μm. Scanned images were then reconstructed into 3D image stacks (NRecon software, Kontich, Belgium) with smoothing 2, beam hardening 20%, ring artifact 5, and a dynamic image range with minimum and maximum attenuation coefficients of 0 and 0.75. They were then analyzed using CT Analyzer software (Skyscan, Kontich, Belgium). The region of interest was the entire fracture callus determined by manual and automated techniques. The proximal and distal boundaries of the callus were identified manually. Next, a custom script was utilized to trace the fracture callus excluding the cortical bone and marrow areas. Bone was differentiated from non-bone by a single global threshold grayscale index of 90. Recorded measures included: tissue mineral density (TMD), bone volume (BV), tissue volume (TV), bone volume/tissue volume (BV/TV), trabecular thickness (Tb.Th), trabecular separation (Tb.Sp), and trabecular number (Tb.N).

### Histomorphometry

Femora harvested for histomorphometric analysis were decalcified in Immunocal™ Decalcifier (StatLab Medical Products, Inc.) at pH 7.2-7.4 for 48 hours. Longitudinal paraffin sections of 5 μm thickness were prepared and the center-most section from each femur was stained with picrosirius red and alcian blue. Brightfield and polarized images were obtained from a Leica DM2700M microscope. Histomorphometric measures of total callus area, cartilage area, fibrous tissue area, and bone area were performed in a blinded fashion using an image manipulation program (BIOQUANT Image Corporation, Nashville, TN) (14).

### Biomechanical analysis

For femora undergoing biomechanical analyses, frozen femora were rehydrated in Dulbecco’s phosphate-buffered saline (DPBS) with calcium and magnesium at room temperature for 16-24 hours. Femora underwent immediate biomechanical torsion analysis after μCT to prevent repeated freeze-thaw cycles and to maintain structural integrity of the bone. Femora were potted into 0.38 bullet casings and held in place with orthodontic resin (Dentsply Sirona, York, PA). The resin was allowed to harden for 5 minutes prior to testing. Biomechanical testing was then performed in torsion at 1 degree/second until failure using a Mark-10 advanced torque-testing system (Copiague, NY). Ultimate torque, stiffness, toughness, and twist to failure were calculated from the resultant torque-twist curves. Ultimate torque was defined as the maximum torque sustained, manual stiffness was defined as the slope of the torque-twist curve over 50-75% of the ultimate torque, maximal stiffness was defined as the greatest slope over a 5 degree segment of twist, toughness was calculated as the area under the torque-twist curve, and twist to failure was defined as the twist to failure after 25% of the ultimate torque was reached on the torque-twist curve (3). The 25% displacement adjustment to the twist to failure was utilized to nullify the initial angular displacement before significant levels of torque were achieved. All fractured femurs were compared to contralateral femurs from the same mouse. A percentage value of the surgical femur as compared to the contralateral femur was obtained for each biomechanical metric.

### Statistics

Continuous variables were summarized by means and standard deviations (SDs). Gwet’s AC2 was calculated to quantify the interrater reliabilities of mRUST scores. The average mRUST scores across raters were then used for subsequent analysis. Spearman’s correlation coefficients were calculated between the mRUST scores and other measures because these associations appeared to be monotonic but nonlinear. For the temporal trend of mRUST scores, a nonlinear mixed effects model was fit with a log-logistic curve as the mean function as follows:


$$Y=Y\left(0\right)-\frac{Y_{\infty }-Y\left(0\right)}{1+exp\left[-k\left\{log\left(X\right)-log\left(X_{0.5}\right)\right\}\right]}$$

where *Y*(0) a known initial value at day zero (mRUST=4 by definition), *Y*_*∞*_ is the upper asymptote at the end of the fracture healing, *k* is the healing rate, and *X*_0.5_ is when half the healing is complete. The log-logistic curve also has an inflection point where the fastest healing happens. Random effects of INLINE, k, and *X*_0.5_ were included. All analyses were performed using SAS 9.4 (SAS Institute, Cary, NC, USA), and p<0.05 was considered statistically significant.

## Results

### mRUST scoring

**Figure 2:**
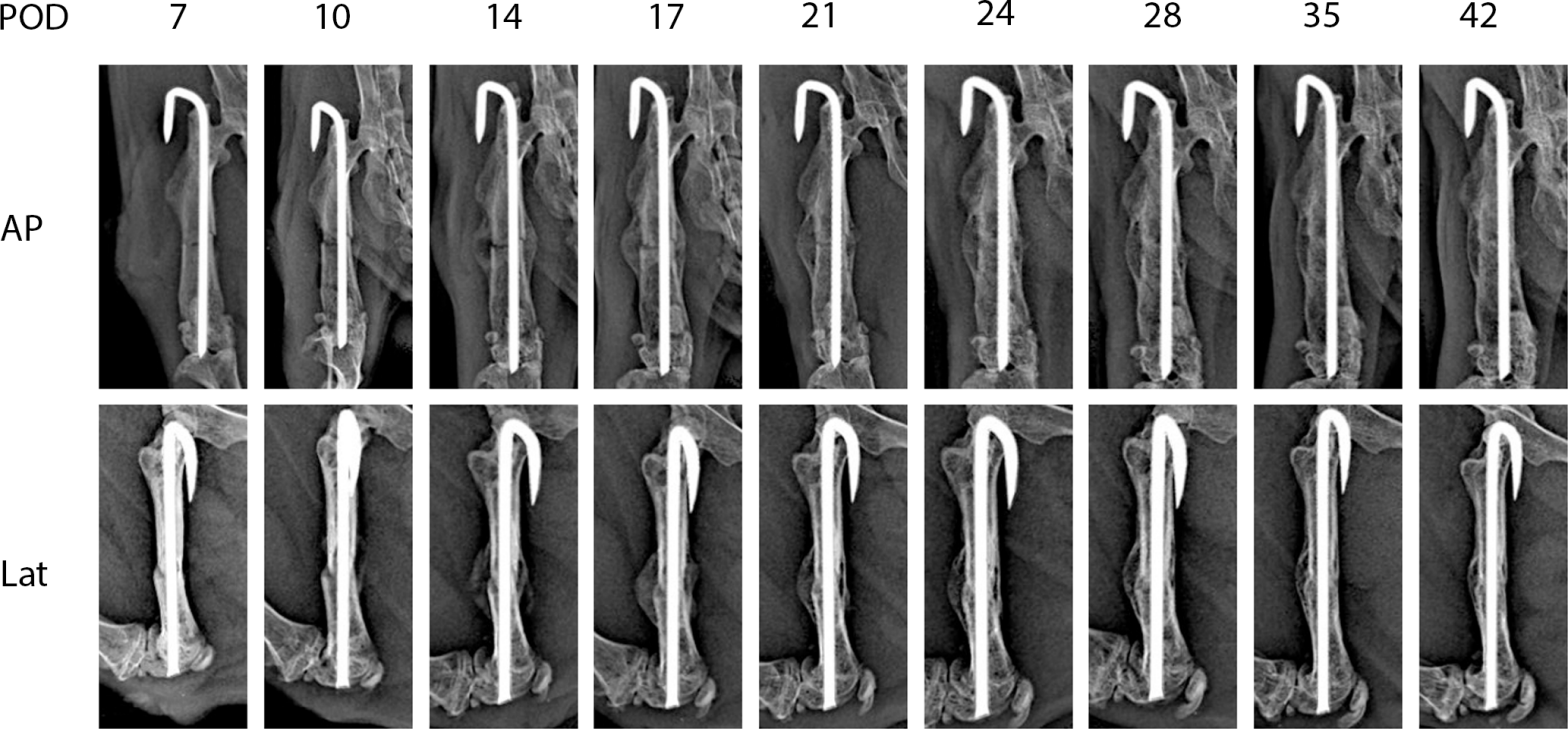
Representative X-rays from each time point used for mRUST scoring. AP, anteroposterior; Lat, lateral; POD, post-operative day.

**Figure 3:**
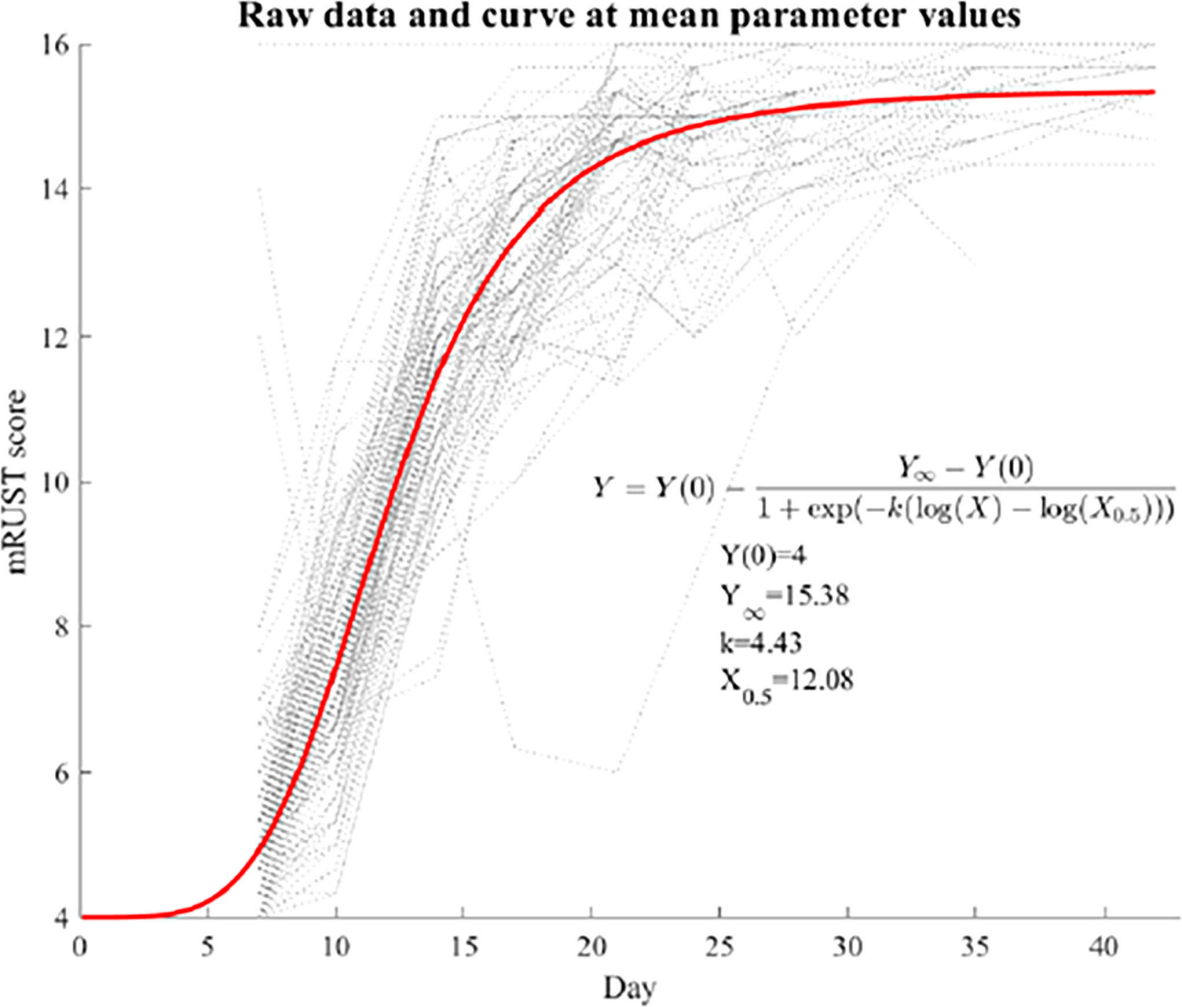
Fit log-logistic curve based on average mRUST scores over time. The underlying dotted curves represent each individual mouse’s healing trajectory.

**Figure 2** shows representative X-ray imaging, both AP and lateral views, at each time point. **Table 1** shows the mean ± SD mRUST scores and interrater reliabilities at each timepoint. Gwet’s AC2 values range from 0.51 to 0.86 indicating substantial interrater reliability. mRUST scores are plotted in **Figure 3**, which also shows the log-logistic regression fit of the data. The three parameters of this sigmoidal curve were estimated yielding the upper asymptote (Y_∞_) as 15.38, the inflection point (X_0.5_) as 12.08 days, and the rate (*k*) as 4.43. Additionally, this curve has approximate exponential growth, transitional, and plateau phases beginning at 7-, 17-, and 28-days post injury, respectively, which reflects the expected timeline of fibrocartilaginous callus, bony callus, and remodeling phases of fracture healing in mice.

**Table 1:** mRUST scores and Gwet’s AC2 interrater analysis ratings based on postoperative day.

mRUST Interrater Correlations		
Post Operative Day	mRUST Score*	Gwet’s AC2**
**7**	5.2 ± 1.7	0.86 [0.80-0.92]
**10**	7.4 ± 1.7	0.63 [0.55-0.71]
**14**	11.7 ± 2.0	0.65 [0.53-0.77]
**17**	13.0 ± 2.0	0.69 [0.59-0.80]
**21**	14.3 ± 2.0	0.62 [0.56-0.68]
**24**	14.6 ± 1.8	0.62 [0.51-0.73]
**28**	14.9 ± 1.6	0.51 [-.39-0.64]
**35**	15.3 ± 1.1	0.67 [0.56-0.78]
**42**	15.5 ± 1.0	0.71 [0.56-0.86]

*Values are Mean ± Standard Deviation, **Values are coefficient [95% Confidence Interval].

### mRUST correlations to traditional measures of fracture repair

**Figure 4:**
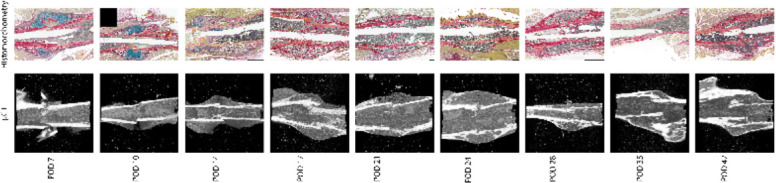
Representative histologic and μCT images for each time point demonstrating the expected progression of fracture healing from soft callus (i.e., cartilaginous on days 7, 10, and 14), to hard callus (i.e., ossification on days 17, 21, and 24), to bony remodeling (days 28, 35, and 42).

**Figure 5:**
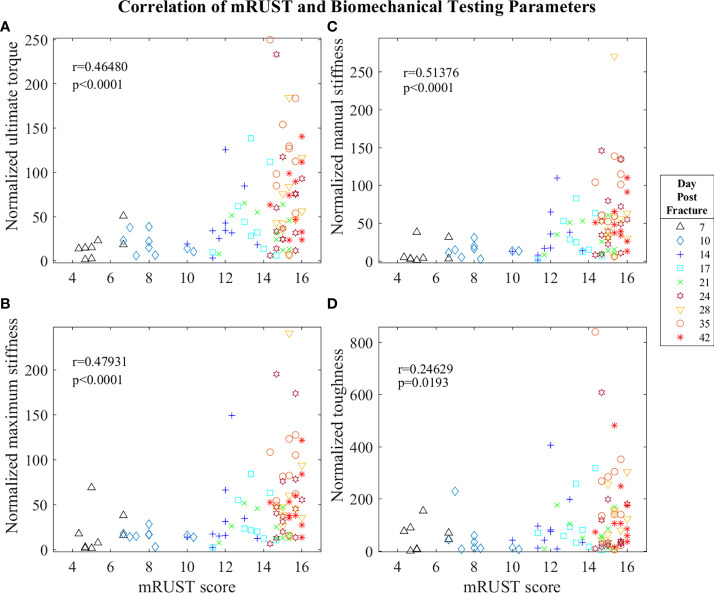
Symbol and color plot of mRUST correlations with **(A)** ultimate torque (% of contralateral), **(B)** maximum stiffness (% of contralateral), **(C)** manual stiffness (% of contralateral), and **(D)** toughness (% of contralateral). Each point represents an individual mouse.

**Figure 6:**
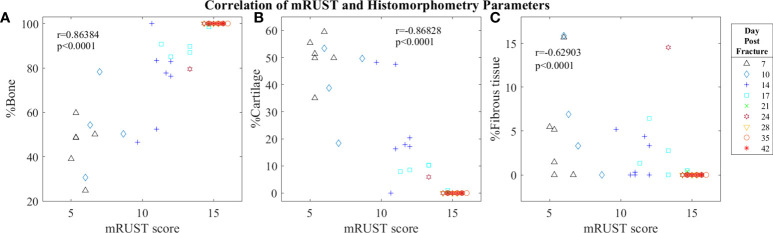
Symbol and color plot of mRUST correlations with **(A)** %Bone, **(B)** %Cartilage, and **(C)** %Fibrous tissue. Each point represents an individual mouse.

**Figure 7:**
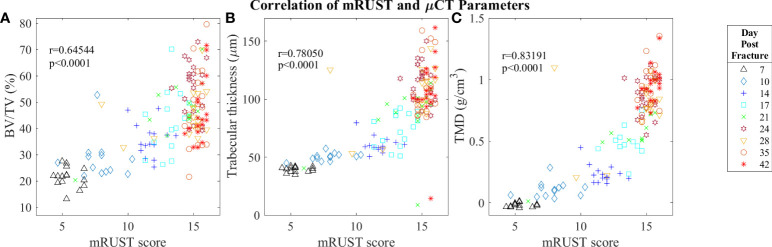
Symbol and color plot of mRUST correlations with **(A)** BV/TV (%), **(B)** Trabecular thickness (μm), and **(C)** Tissue Mineral Density (g/cm)^3^. Each point represents an individual mouse. .

For mRUST to be a viable metric of murine fracture healing, it should correlate well with traditional measures. **Figure 4** shows representative histologic and μCT images for each time point. **Figures 5**–**7** display scatter plots of select traditional measures of fracture repair that correlated well with mRUST scores. Correlations of mRUST with other measures are listed in **Table 2**.

**Table 2:** Spearman correlation coefficients of mRUST and other fracture healing parameters over time.

Standard Measures of Fracture Repair	Correlation to mRUST (Spearman coefficient, p-value)
*Biomechanics*	
Secant Stiffness* (% of contralateral)	0.38828, 0.0002
Twist to Failure (% of contralateral)	-0.30323, 0.0037
*Histomorphometry*	
Bony area (μm^2^)	-0.01465, 0.9204
Cartilage area (μm^2^)	-0.84318,<.0001
Fibrous tissue area (μm^2^)	-0.62686,<.0001
Total area (μm^2^)	-0.38540, 0.0062
*μCT*	
Tissue volume (TV, μm^3^)	-0.02602, 0.7544
Bone volume (BV, μm^3^)	0.15047, 0.0689
Trabecular separation (μm)	0.45434,<.0001
Trabecular number	-0.47287,<.0001

*Secant stiffness: slope of the line between 0 and the maximum torque of the torque-twist relationship.

Biomechanical testing offers the most direct quantification of bone’s ability to support efficient locomotion and is thus pivotal for determining the functionality of the end product of fracture repair. As observed in **Figure 5**, we found mRUST to correlate with the biomechanical testing measures ultimate torque (r=0.46, p<0.0001), manual stiffness (r=0.51, p<0.0001), maximal stiffness (r=0.48, p<0.0001), and toughness (r=0.25, p=0.0193). The direction of all these correlations are such that as mRUST scores increase, so do these biomechanical parameters. This is expected, as the bones should become more biomechanically sound as they progress in fracture healing.

Histological staining followed by histomorphometry offers another avenue for assessment of the fracture callus that is best for differentiating between tissue types (cartilage, fibrous, bone, and marrow). As detailed in **Figure 6**, we found mRUST to also correlate with the histomorphometry measures bone percentage (r=0.86, p<0.0001), cartilage percentage (r=-0.87, p<0.0001), and percentage fibrous tissue (r=-0.63, p<0.0001). The direction of the correlations to mRUST are positive for bone percentage and negative for cartilage and fibrous tissue percentage. This is also expected as it corresponds to normal fracture healing, which proceeds through stages from inflammation/hematoma, to fibrocartilaginous callus, to bony callus, to remodeling. Thus, as more bone healing is seen on X-ray (increased mRUST), bone percentage should increase while cartilage and fibrous tissue percentages decrease.

Micro-computed tomography enables high‐resolution visualization of fracture callus architecture, and resolution of fracture related deficits in μCT parameters often correlates with restoration of mechanical function. As shown in **Figure 7**, we found mRUST to correlate well with the micro-computed testing measures TMD (r=0.83, p<0.0001), BV/TV (r=0.65, p<0.0001), and trabecular thickness (r=0.78, p<0.0001). The direction of the correlations to mRUST are positive for TMD, BV/TV, and trabecular thickness. This is again expected since, as more bone healing is seen on X-ray (increased mRUST), there should be more bone on μCT, which corresponds to increased BV/TV and trabecular thicknesses. μCT also showed that from day 24 onwards all specimens were healed with callus bridging all four cortices and fracture line remaining evident only on two or fewer cortices, which corresponds well to the observed radiograph derived mRUSTs >14 at these same time points.

## Discussion

The goal of this study was to assess the validity of mRUST X-ray scoring as a metric of fracture repair outcomes in mice. X-ray evaluation is pivotal in gauging fracture repair success clinically. In fact, the FDA defines non-union solely based on radiographic criteria and definitions of non-union in orthopaedic research include radiographic criteria more often than clinical criteria (62% vs 45%) (15). Several clinical studies have associated RUST or mRUST scoring with nonunion risk (11, 16–18) and with good interrater reliabilities (11, 18). Similar to these clinical studies, our results show that mRUST scoring of murine femoral fractures can quantify fracture healing over time with substantial interrater reliability. It should be noted that mRUST scoring is commonly used as a “binary” measure clinically, with scores above a threshold being defined as healed and below as not healed. The log-logistic analysis of our murine mRUST data is more likely beneficial to model drugs that improve healing or disease states that are detrimental to healing from a normal profile, and not necessarily useful to predict which fractures will heal versus not heal.

Despite the known predictive value and reliability of RUST/mRUST scoring of fracture repair success, there is minimal evidence supporting its use in animal models (4, 19–22). Our current study implemented mRUST scoring alongside μCT, histomorphometric, and biomechanical assessments to adequately characterize mRUST trends and its correlation to established metrics of fracture repair in mice. To our knowledge, one similar study in mice (4) and two similar studies in rats have previously been reported (19, 23). Interestingly, comparison of μCT, histomorphometric, and biomechanical parameters to mRUST scoring appears to have a biphasic relationship in our study, whereby once a mRUST score of 14 is reached there is considerable clustering of the various parameters (see **Figures 5**-**7**).

In the study by Cooke and colleagues (4), the authors utilized male, C3H/HeJ mice at 12 weeks of age. Half of the mice were given no phosphate for 2 days prior to fracture induction until 17 days post-fracture. Terminal μCT was assessed at post-fracture day 14, 21, 35, and 42. mRUST scores were positively correlated with callus bone mineral density (BMD) (r=0.85; p<0.001) and bone volume fraction (i.e., BV/TV) (r=0.86; p<0.001) and negatively correlated with total callus volume (r=-0.54; p<0.001). Significant but weaker correlations were found between the mRUST scores and the mechanical properties of callus strength (r=0.45; p<0.02), stiffness (r=0.52; p<0.001), and rigidity (r=0.5; p<0.001), and negatively correlated to twist to failure (r=-0.37; p<0.001). However, there was no correlation with toughness (p>0.5). A few distinctions between our study design may account for differences in findings. We used C57BL6/J mice with no diet modifications. Also, instead of X-ray imaging, the previous authors utilized μCT reconstructed images for mRUST scoring. Further, fractures were created utilizing a guillotine model which has been found to be less consistent than surgical fracture induction with dental wire cutters as we implemented in this study (24). Lastly, we recorded data at several additional timepoints. In our study, the parameters that correlated best with mRUST were histomorphometry measures of bone percentage (r=0.86) and cartilage percentage (r=-0.87), followed closely by tissue mineral density on μCT (r=0.83). As a fracture heals, more bone is seen on X-ray which corresponds to the positive correlations for mRUST with bone percentage and trabecular thickness, as well as to the negative correlation of percent cartilage as the cartilage becomes ossified. While the study by Cooke and colleagues (4) showed higher correlation of mRUST scoring with μCT BV/TV compared to our study, correlation to callus mineral density was similar. It is possible that μCT could be more reliable in preclinical studies to monitor healing over time; however, it should also be noted that the correlation in the study by Cooke and colleagues (4) is between μCT parameters and μCT simulated radiographs, which may increase the correlation compared to correlations determined from distinct methods (i.e., μCT and X-ray).

Fiset et al. (19) performed a femoral osteotomy in a rat model stabilized with plate and screws. Rats were sacrificed at 4-, 5-, 6-, 7-, 8-, 9-, and 17-weeks. Bone volume, total callus volume, and percent bone volume correlated better with mRUST scores than bone mineral density. Significant correlation existed between mRUST and torsional stiffness, ultimate torque, and twist to failure (p<0.001, p<0.001, and p<0.01, respectively). Notably, the authors advocated for an mRUST threshold of 15 to define bony union due the fact that 140% of ultimate torque of the intact femur was achieved at this threshold score. We found that mRUST scores >15 correlated with ultimate torque scores of >50% of the contralateral femur. Further, our μCT showed that from day 24 onwards all specimens were healed, and mRUSTs were >14 at these time points. Finally, in a study of rat tibial fractures at various stages of healing, Tawonsawatruk et al. utilized the RUST score to test the consistency of scoring among assessors (20). There was no mention of technique used to generate the fractures. The authors did demonstrate better inter- and intra-observer consistency with RUST than general impression scoring. However, the mRUST score was not analyzed.

To our knowledge, RUST and mRUST have been used as metrics to examine non-RUST and mRUST related hypotheses in few rodent studies. Meeson et al. (25) implemented RUST to assess whether administration of VEGF with AMD3100 (CXCR4 antagonist) could mobilize MSCs into the peripheral circulation of rats, and to determine whether increasing the circulating levels of MSCs would improve fracture healing in a delayed union rat femoral fracture model. RUST scoring performed 5 weeks post injury showed mean scores of 7.71 ± 2.7 for the control group and 9.63 ± 1.3 for the VEGF-AMD group. The VEGF-AMD group also showed a higher mean bone volume, trabecular thickness, and percent bone volume than controls on μCT, and significantly greater number of mesenchymal stem cell colony forming units/ml compared to controls. In another study, Cahill et al. (26) implemented RUST to assess the impact of MRSA on fracture repair and the efficacy of rifampin treatment in ameliorating repair deficits. On post-operative day 28 mean RUST scores were 11.0, 6.0 and 10.7 in the no infection, untreated infection, and combination local and systemic rifampin groups, respectively. These unique experiments demonstrate RUST scoring can elucidate differences between experimental groups.

We predict that in studies with mRUST scoring at sufficient timepoints, the log-logistic curve fit shown in **Figure 3** will be useful in characterizing differences in fracture healing outcomes following various experimental interventions. For instance, we can hypothesize what expected results following bisphosphonate or RANKL-inhibitor administration might look like. These agents have been shown to decrease resorption of mineralized cartilage and mineralized bone throughout repair, resulting in higher percent bone volumes and increased union, at the expense of ultimate fracture repair quality with increased unresorbed cartilage (27). Theoretically, this may manifest in increased mRUST scores earlier in fracture repair (due to increased portion of mineralized cartilage and bone) but a decreased plateau (since highest mRUST scores require remodeling which could be visibly decreased). These predictions might be visualized on the log-logistic fit curve as a decreased upper asymptote (Y_∞_), an increased or unchanged rate (*k*), and an earlier inflection point (X_0.5_). Additionally, although mRUST appears to correlate well with other modalities of fracture repair assessment, if and how these correlations may be disrupted in various disease states and following experimental interventions is unclear. For instance, Cooke et al. (4) found that in mice with a phosphate deficient diet, healed fractures exhibited decreased biomechanical parameters but unchanged mRUST scores.

Several limitations to our study exist. We had only three raters and do not know how this would fair with a larger number of raters with varying degrees of experience. Another limitation is that we intended to house five mice per cage. Over the course of the study, some were split due to aggression or other behavior based on veterinarian and/or study personnel observation. This may have contributed to variation in measured parameters as previously described (28). Additionally, though it is common practice to for biomechanical specimens to undergo a single freeze-thaw cycle (29), this is a possible explanation for why biomechanical testing parameters had the lowest correlation with mRUST. Alternatively, biomechanical testing is commonly the most variable measurement for murine fracture healing studies (13). This variability would then be reflected in the correlation analyses. Further, while we had the largest study of mRUST on a murine model to date, larger cohorts of the various fracture healing modalities are needed to more accurately determine their correlation to mRUST values. Future studies should utilize large sample sizes to explore the impact of differences in rater experience, fixation strategies (e.g., pin size), mice age, gender, strains, disease states and other experimental interventions (e.g., pharmaceutical administration). Considering the 3R’s (replacement, reduction, and refinement) of animal welfare, these goals might be accomplished using existing data or by incorporating mRUST scoring as an additional measure in ongoing studies. Finally, we did not compare longitudinal X-ray to other established longitudinal imaging modalities used in rodent fracture healing (e.g., *in vivo* µCT, SPECT). We chose radiographic assessment, as this is the most commonly used clinical modality and is less technically demanding/time intensive compared with other protocols for rodent non-invasive longitudinal monitoring of fracture healing. Our purpose is not to supplant other modalities, but rather to investigate whether quantitative evaluation of mRUST would at all be of use when applied longitudinally to radiographic evaluation. We demonstrate that it is a feasible technique, has reasonable correlations to standard measures of rodent fracture healing, and we uniquely provide a “mathematical” description of routine fracture healing based on mRUST scoring over time that could be valuable for future studies. X-rays may also be useful in cases where the more advanced imaging modalities are not available.

In conclusion, mRUST is a reliable, cost-effective, and non-terminal measure to follow fracture healing longitudinally in murine models. It follows a consistent trend that reflects the phases of fracture repair and correlates well with traditional metrics of fracture repair. We predict that implementation of the log-logistic mRUST curve will enable accurate and meaningful representation of various alterations in fracture repair following experimental intervention. This will facilitate characterization of healing trajectory in endocrine disease states, such as menopausal osteoporosis, glucocorticoid use, renal osteodystrophy, pituitary disease, and anorexia nervosa, as well as assess the efficacy of potential treatments. For large exploratory studies (e.g., drug testing), a shift in parameters and phases of the log-logistic curve could be used to gauge general impact on fracture healing. Upon finding a change in mRUST healing trajectory, more detailed experiments involving μCT, histomorphometry, biomechanics and other assessments could be undertaken at targeted time points. In this way, longitudinal mRUST trajectories could expedite discovery of new fracture healing interventions for further investigation.

## Data availability statement

The original contributions presented in the study are included in the article/supplementary material. Further inquiries can be directed to the corresponding author.

## Ethics statement

The animal study was reviewed and approved by Indiana University School of Medicine Institutional Animal Care and Use Committee.

## Author contributions

RN, MK, CC and T-MC conceived of the idea, designed the experiments and assisted with data interpretation and manuscript preparation. MK provided equipment, funding and other resources. VA, AK, CS, AM, JC, SM, MS, RB, UD, CC, JL and RN participated in conducting experiments and acquisition of data. ZL curated data and performed formal analyses. VA and AM drafted original manuscript. All authors assisted in editing the manuscript, approve of the final version, and take responsibility for the accuracy and integrity of the work.

## Funding

Funding for these studies was provided in part by the NIH (AR065971, HK110854, U54 DK106846, AG060621). This work was also supported in part by Indiana University School of Medicine, the Indiana Center for Musculoskeletal Health, the Stark Neuroscience Research Institute, and the Department of Orthopaedic Surgery. This material is also the result of work supported with resources and the use of facilities at the Richard L. Roudebush VA Medical Center, Indianapolis, IN: VA Merit #BX003751. The presented contents are solely the responsibility of the authors and do not necessarily represent the official views of any of the aforementioned agencies.

## Conflict of interest

The authors declare that the research was conducted in the absence of any commercial or financial relationships that could be construed as a potential conflict of interest.

## Publisher’s note

All claims expressed in this article are solely those of the authors and do not necessarily represent those of their affiliated organizations, or those of the publisher, the editors and the reviewers. Any product that may be evaluated in this article, or claim that may be made by its manufacturer, is not guaranteed or endorsed by the publisher.
